# Supplementary material for: Discovery of atomic clock-like spin defects in simple oxides from first principles
Source: Nat Commun. 2024 Jun 6;15:4812. doi: 10.1038/s41467-024-49057-8 (PMC11156963; doi:10.1038/s41467-024-49057-8)
Supplement: Supplementary file 1 — Supplementary Info [file 41467_2024_49057_MOESM1_ESM.pdf]

# SUPPLEMENTARY INFORMATION:
# Discovery of Atomic Clock-Like Spin Defects in Simple Oxides from First Principles

Joel Davidsson,[1,*] Mykyta Onizhuk,[2,†] Christian Vorwerk,[2] and Giulia Galli[3,4,‡]

[1]*Department of Physics, Chemistry and Biology, Linköping University, SE-581 83 Linköping, Sweden*

[2]*Pritzker School of Molecular Engineering, University of Chicago, Chicago, Illinois 60637, United States*

[3]*Pritzker School of Molecular Engineering and Department of Chemistry, University of Chicago, Chicago IL 60637, USA*

[4]*Materials Science Division and Center for Molecular Engineering, Argonne National Laboratory, Lemont IL 60439, USA*

[*] joel.davidsson@liu.se

[†] onizhuk@uchicago.edu

[‡] gagalli@uchicago.edu

## SUPPLEMENTARY NOTE 1. POTENTIAL ENERGY SURFACE FOR P AND AS DOPANTS

As mentioned in the main text, in the case of P and As dopants, we found two local minima in the total energy of the system, along the path connecting the positions of $V_{Ca}$ and $V_O$. Depending on whether the dopant is initially placed at the Ca or O site, we find different local minima when we optimize the total energy. In the two relaxed structures, the position of the dopants differs by 0.54 Å for P and 0.52 Å for As, see Supplementary Figure 1. Interestingly, for P and As, only the minima closest to the Ca site have the same electronic structure as that of Sb, Bi, and I. These minima correspond to the lowest energy structures, with a difference of 74 meV for the P dopant and 283 meV for the As dopant relative to the metastable minima. However, for the P and As dopants, we could not obtain a satisfactory convergence in the excited states, and due to these difficulties, we omitted these defects from our discussion and focused on the Sb, Bi, and I dopants instead, where only one minimum is found regardless of the starting point of the total energy minimization, and excited states could be properly converged.

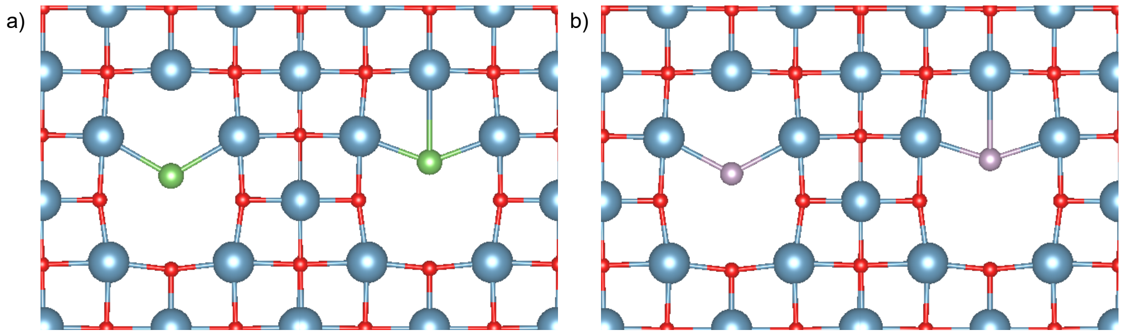


Supplementary Figure 1. **The two different minima for the P and As defects.** The left image shows the minimum closest to the Ca site ($V_{Ca}$) and right image the minimum closest to the O site ($V_O$).

## SUPPLEMENTARY NOTE 2. FORMATION ENERGY OF DEFECTS USING SEMI-LOCAL AND HYBRID FUNCTIONALS

The formation and binding energies of the Sb, Bi, and I dopants are plotted in Supplementary Figure 2 with the PBE, HSE, and tuned HSE functional. Compared with the PBE results (obtained from ADAQ), the band gap is increased when using HSE06, thus increasing the region of stability for the charge state with spin-1 for all defects considered here. This range of stability is further increased when tuning the short-range Hartree-Fock exchange (more details on the tuned HSE functional are given in the next section). These formation energies are reported with only reference chemical potentials.

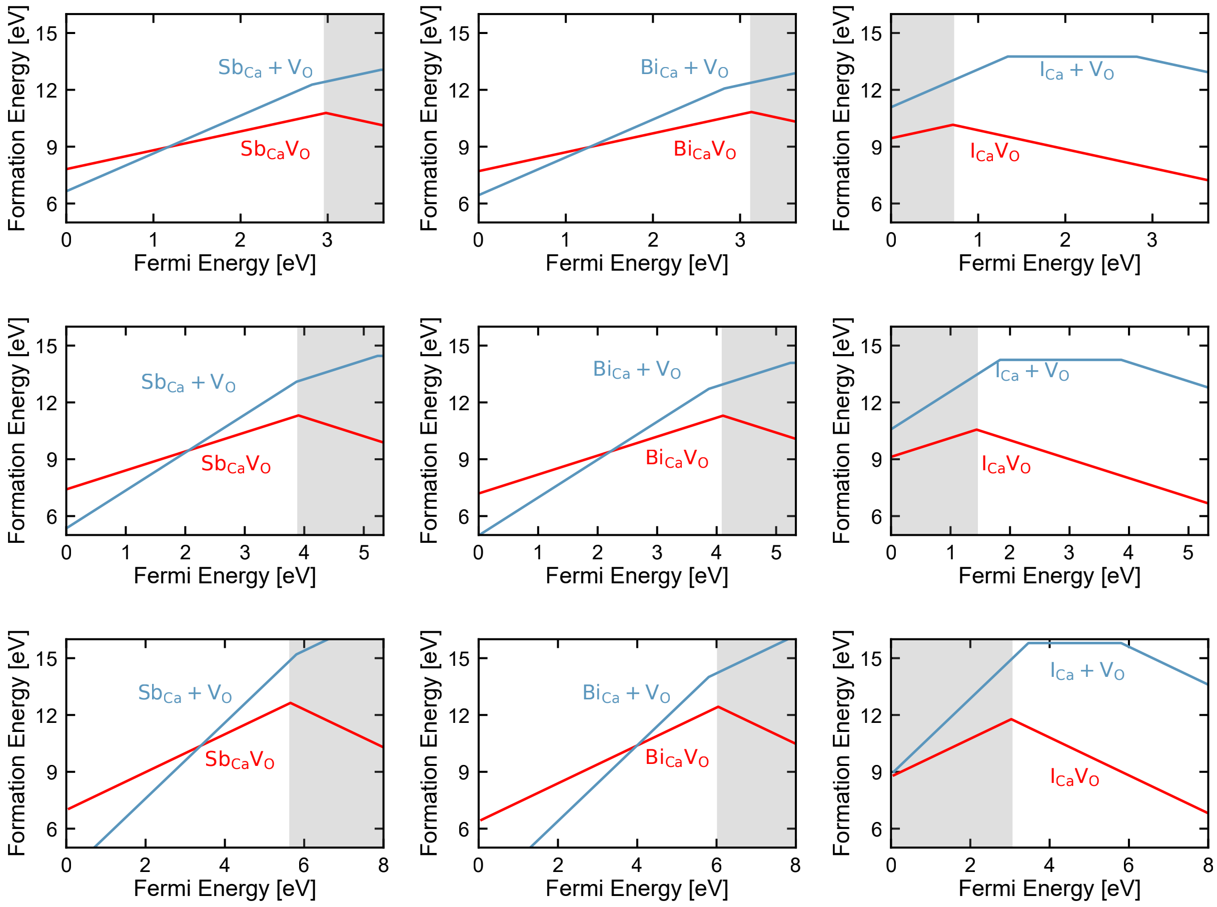


Supplementary Figure 2. **Formation energies with semi-local and hybrid functionals.** Formation energy of the $X_{Ca}V_O$ defects, where $X = Sb, Bi, I$. The first row shows the PBE results from ADAQ, the second row shows the HSE06 results, and the third row show the HSE06 results with with 62.5% short-range Hartree-Fock exchange. The shaded regions shows the region of stability for the spin-1 state.

## SUPPLEMENTARY NOTE 3. TUNED SHORT-RANGE HARTREE-FOCK EXCHANGE OF THE HSE06 FUNCTIONAL

Since the HSE06 functional underestimates the band gap of CaO, we tuned the short-range Hartree-Fock exchange to match the experimental band gap following the procedure outlined in Ref. 1. The conventional unit cell was relaxed using a $3 \times 3 \times 3$ gamma-centered Monkhorst-Pack k-point mesh. A short-range Hartree-Fock exchange of 62.5% gives a lattice parameter of 4.738 Å and a band gap of 7.465 eV. Increasing the k-point mesh to $5x5x5$ only changes the band gap by 0.01 eV. By subtracting the zero-point renormalization (0.34 eV [2] or 0.36 eV [3]), the band gap is 7.125 or 7.105 eV, a value close to the experimental band gap of 7.09 eV [4]. The Sb, Bi, and I complexes were also investigated with the tuned HSE functional in a $4 \times 4 \times 4$ supercell with the gamma point only; the results are presented in Supplementary Figure 2. In the following subsections, we discuss different charge corrections and how the choice of chemical potentials affects the stability of single defects as well as growth conditions.

### A. Charge Corrections in DFT Calculations

As mentioned in the main text, to obtain the high-throughput results with the PBE functional, we used the Lany-Zunger (LZ) charge correction for all defects. The correction $E_{\text{corr}}$ to the total energy is:

$$E_{\text{corr}} = (1+f)\frac{q^2\alpha_M}{2\epsilon L}, \tag{1}$$

where $(1+f)$ is set to 0.65 [5], $q$ is the defect charge, $\alpha_M$ is the Madelung constant that is set to 2.8373 for simple cubic crystals, $\epsilon = 4\pi\epsilon_0\epsilon_r$ where $\epsilon_r$ is the dielectric constant (11.95 for CaO), and $L$ is the length of the supercell (19.315 Å in our PBE simulations). With these values, we obtain $E_{\text{corr}} = 0.058$*q$^2$ eV.

For calculations with the HSE functionals, we used the Freysoldt-Neugebauer-Van de Walle (FNV) correction [6, 7]. Table S1 shows the values of the total energy corrections for the defects considered in our calculations. The FNV corrections are close to the LZ correction of 0.058 eV for single charges and 0.232 eV for double charges. Hence, using the FNV instead of LZ correction amounts to a negligible difference between the computed charge transition levels, due to the large supercell used here and the small values of the defect charge q ($q = \pm1$ and $q = \pm2$) considered in our study.

TABLE S1. The FNV correction for the defects in CaO calculated with the HSE functional. C is the long-range potential shift and $E_{\mathrm{corr}}$ is the correction including screening and alignment.

| Defect | charge | C [eV] | $E_{\mathrm{corr}}$ [eV] |
|---|---|---|---|
| $Sb_{Ca}V_O$ | + | 0.02 | 0.110193 |
| $Sb_{Ca}V_O$ | - | 0.03 | 0.0601929 |
| $Sb_{Ca}$ | + | 0.015 | 0.105193 |
| $Bi_{Ca}V_O$ | + | 0.03 | 0.120193 |
| $Bi_{Ca}V_O$ | - | 0.03 | 0.0601929 |
| $Bi_{Ca}$ | + | 0.02 | 0.110193 |
| $I_{Ca}V_O$ | + | 0.01 | 0.100193 |
| $I_{Ca}V_O$ | - | 0.0 | 0.0901929 |
| $I_{Ca}$ | + | 0.015 | 0.105193 |
| $I_{Ca}$ | - | 0.015 | 0.0751929 |
| $V_O$ | 2+ | -0.02 | 0.320772 |
| $V_O$ | + | -0.015 | 0.0751929 |
| $V_{Ca}$ | 2- | -0.01 | 0.380772 |
| $V_{Ca}$ | - | -0.015 | 0.105193 |
| $O_{Ca}$ | 2- | 0.01 | 0.340772 |
| $O_{Ca}$ | - | 0.01 | 0.0801929 |
| $Ca_O$ | 2+ | -0.01 | 0.340772 |
| $Ca_O$ | + | -0.01 | 0.0801929 |
| $Int_O$ | 2- | -0.04 | 0.130193 |
| $Int_O$ | - | -0.01 | 0.100193 |
| $Int_{Ca}$ | 2+ | 0.0 | 0.360772 |
| $Int_{Ca}$ | + | 0.01 | 0.100193 |

### B. Doping and Growth Conditions of CaO

In oxides, single point defects can have negative formation energy, thus restricting the desired range of Fermi level doping to the region where these defects are stable [8]. In our high-throughput calculations, we considered various single point defects: $V_{Ca}$, $V_{O}$, $Int_{Ca}$, $Int_{O}$, $Ca_{O}$, and $O_{Ca}$. Here, V stands for vacancy, and Int for interstitial. These defects, up to double charge states, were investigated with the tuned HSE functional with 62.5% short-range Hartree-Fock exchange.

The chemical potential is defined as $\mu = \mu_{\mathrm{ref}} + \Delta\mu$, where $\mu_{\mathrm{ref}}$ is either the energy of Ca bulk or that of the $O_2$ molecule. The $\Delta\mu$ is bounded by the formation enthalpy of CaO ($\Delta H_f(\mathrm{CaO})$) that is -6.35 eV, which agrees well with other theoretical results (-6.15 eV in Ref. [9]) and the experimental value -6.58 eV [10]. Hence, the two conditions are O-rich ($\Delta\mu_{\mathrm{O}} = 0$ and $\Delta\mu_{\mathrm{Ca}} = \Delta H_f(\mathrm{CaO})$) and Ca-rich ($\Delta\mu_{\mathrm{O}} = \Delta H_f(\mathrm{CaO})$ and $\Delta\mu_{\mathrm{Ca}} = 0$) [11]. Supplementary Figure 3 shows the single defects in O- or Ca-rich conditions.

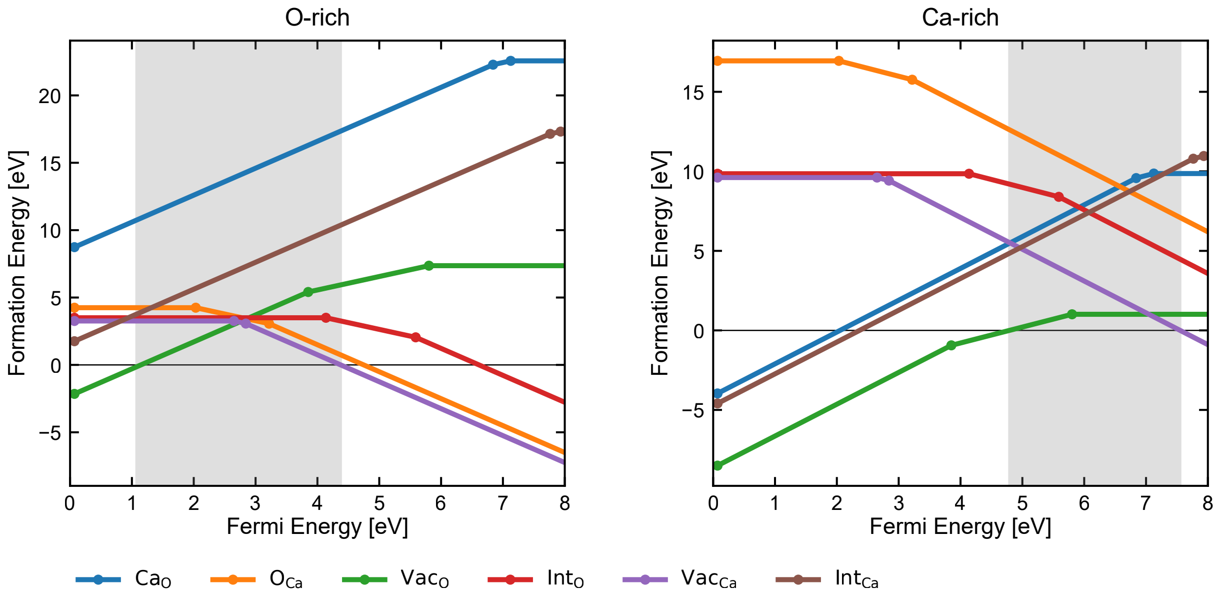


Supplementary Figure 3. **Chemical potential effects on formation energies.** The formation energy of single point defects in CaO calculated with the tuned HSE functional with 62.5% short-range Hartree-Fock exchange for O- or Ca-rich conditions. The shaded regions shows the Fermi energy range where single defects do not spontaneously form.

Supplementary Figure 3 shows the range of Fermi level values where no single defect has negative formation energy. This range is different, depending on the growth conditions: O- or Ca-rich conditions. Our results show that Ca-rich conditions are required to stabilize the Sb and Bi defects in the negative charge state, which has spin-1. Instead, O-rich conditions are required for the I defect to stabilize the positive charge with spin-1. Since the spin-defects proposed here consist of an oxygen and calcium vacancy, their formation energy will decrease with $\Delta H_f(\text{CaO})$, regardless of dopants. Supplementary Figure 3 in the main text shows the formation energy and optimal growth conditions for each defect.

## SUPPLEMENTARY NOTE 4. GROUND STATE PHONONS

For the $Bi_{Ca}V_O{}^-$ defect, we calculated the phonons of the whole system using phonopy [12, 13], where we enforced a $C_{4v}$ symmetry for the defect. In the 511 atoms supercell, there were 478 displacements, for which the calculations were carried out with the PBE functional with the same settings as specified in the main text, except that the convergence condition of the total energy was set to $10^{-8}$ eV and the projection operators were evaluated in reciprocal space. Supplementary Figure 4 shows the inverse participation ratio (ipr) of the phonon eigenmodes as a function of the phonon frequency.

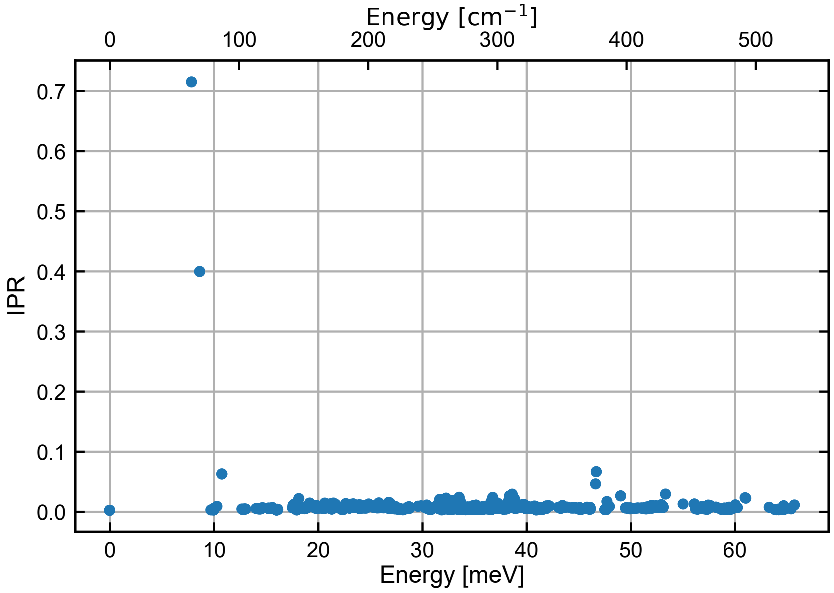


Supplementary Figure 4. **Ground state phonons.** Computed values of the inverse participation ratio (IPR) of the phonon modes as a function of the phonon energy, for a CaO cell with 511 atoms, containing the $Bi_{Ca}V_O{}^-$ defect.

We find that the most localized mode (at 7.8 meV with ipr of 0.71) in the system is mainly localized on the Bi atom and is likely to be a rattling mode. The next highest localized mode (at 8.6 meV and ipr of 0.40) is degenerate, and corresponds to an $e$ mode.

## SUPPLEMENTARY NOTE 5. PHOTOLUMINESCENCE SPECTRA

We computed photoluminescence (PL) spectra using the method outlined in Ref. 14. The partial Huang-Rhys factors were extracted with pyphotonics [15] using the ground state geometry, computed phonons and excited state geometry. With the PBE functional, the Jahn-Teller splitting between the symmetry-constrained excited state (with half-half occupation) and symmetry-broken excited state (with single occupation) is about 0.1 meV. Hence, we used the symmetry-constrained excited state geometry as often adopted for the NV center in diamond [14].

When considering all phonons, the Huang-Rhys (HR) factor is 19.55 for the $Bi_{Ca}V_O{}^-$ defect. However, this large factor may possibly be decreased with nanostructuring, as shown, e.g., in the case of the silicon vacancy in SiC [16], where the Debye-Waller (DW) factor was increased from 6% in bulk to 58% in nanowires. If we assume that reducing the contributions of the bulk phonons is responsible for this improvement, we can do the same for the defect in CaO. if we discard phonons with an ipr below 0.01, we obtain a HR factor of 1.11 (corresponding to a DW factor of 33%). Both spectra with all phonons modes and only localized modes shown in Supplementary Figure 5. Further calculation with better functional and experimental validation are needed to obtain a robust strategy to improve the DW factor.

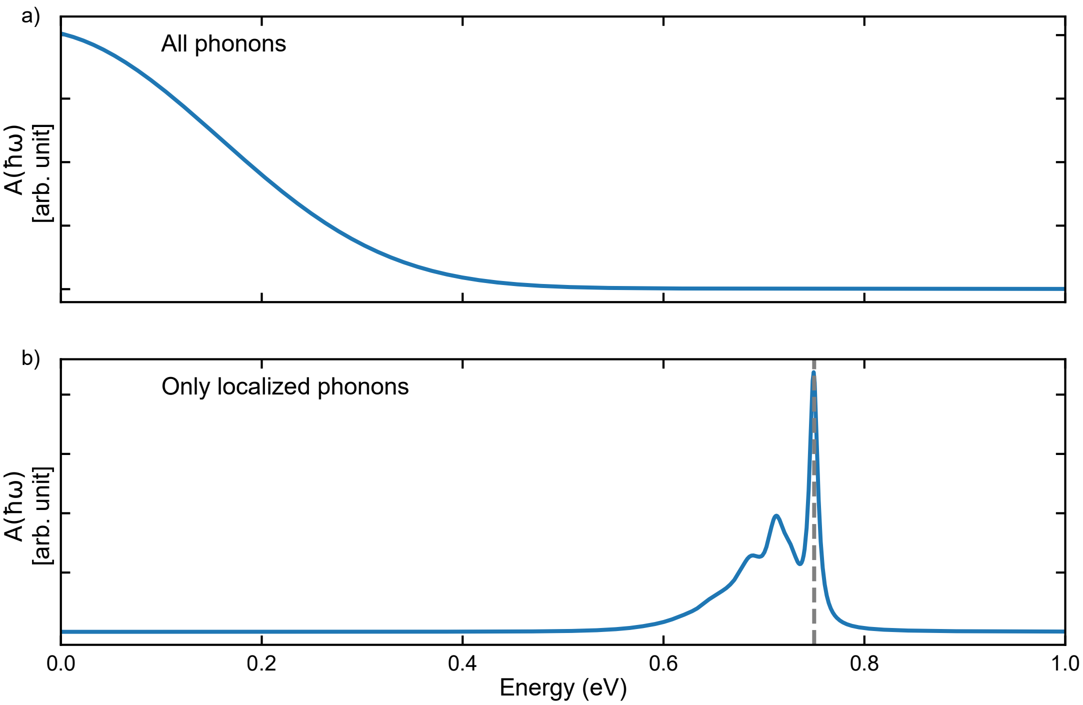


Supplementary Figure 5. **Simulated photoluminescence spectra.** The emission spectra of the $Bi_{Ca}V_O{}^-$ defect in CaO with a) all phonon modes and b) only phonons with ipr above 0.01. ZPL and phonon broadening parameters are both set to 5 meV.

## SUPPLEMENTARY REFERENCES


1. Chen, Y., Turiansky, M. E. & Van de Walle, C. G. First-principles study of quantum defect candidates in beryllium oxide. *Phys. Rev. B* **106,** 174113. `https://link.aps.org/doi/10.1103/PhysRevB.106.174113` (17 Nov. 2022).

2. Miglio, A. *et al.* Predominance of non-adiabatic effects in zero-point renormalization of the electronic band gap. *npj Computational Materials* **6,** 167. ISSN: 2057-3960. `https://doi.org/10.1038/s41524-020-00434-z` (Nov. 2020).

3. Engel, M. *et al.* Zero-point renormalization of the band gap of semiconductors and insulators using the projector augmented wave method. *Phys. Rev. B* **106,** 094316. `https://link.aps.org/doi/10.1103/PhysRevB.106.094316` (9 Sept. 2022).

4. Whited, R., Flaten, C. J. & Walker, W. Exciton thermoreflectance of MgO and CaO. *Solid State Communications* **13,** 1903–1905. ISSN: 0038-1098. https://www.sciencedirect.com/science/article/pii/0038109873907540 (1973).

5. Lany, S. & Zunger, A. Assessment of correction methods for the band-gap problem and for finite-size effects in supercell defect calculations: Case studies for ZnO and GaAs. *Phys. Rev. B* **78,** 235104. http://link.aps.org/doi/10.1103/PhysRevB.78.235104 (23 Dec. 2008).

6. Freysoldt, C., Neugebauer, J. & Van de Walle, C. G. Fully Ab Initio Finite-Size Corrections for Charged-Defect Supercell Calculations. *Phys. Rev. Lett.* **102,** 016402. https://link.aps.org/doi/10.1103/PhysRevLett.102.016402 (1 Jan. 2009).

7. Freysoldt, C., Neugebauer, J. & Van de Walle, C. G. Electrostatic interactions between charged defects in supercells. *physica status solidi (b)* **248,** 1067–1076. eprint: https://onlinelibrary.wiley.com/doi/pdf/10.1002/pssb.201046289. https://onlinelibrary.wiley.com/doi/abs/10.1002/pssb.201046289 (2011).

8. Robertson, J. & Clark, S. J. Limits to doping in oxides. *Phys. Rev. B* **83,** 075205. https://link.aps.org/doi/10.1103/PhysRevB.83.075205 (7 Feb. 2011).

9. Osorio-Guillén, J., Lany, S., Barabash, S. V. & Zunger, A. Magnetism without Magnetic Ions: Percolation, Exchange, and Formation Energies of Magnetism-Promoting Intrinsic Defects in CaO. *Phys. Rev. Lett.* **96,** 107203. https://link.aps.org/doi/10.1103/PhysRevLett.96.107203 (10 Mar. 2006).

10. Haynes, W. M., Lide, D. R. & Bruno, T. J. *CRC handbook of chemistry and physics* 95th edition (CRC press, 2016).

11. Freysoldt, C. *et al.* First-principles calculations for point defects in solids. *Rev. Mod. Phys.* **86,** 253–305. https://link.aps.org/doi/10.1103/RevModPhys.86.253 (1 Mar. 2014).

12. Togo, A., Chaput, L., Tadano, T. & Tanaka, I. Implementation strategies in phonopy and phono3py. *J. Phys. Condens. Matter* **35,** 353001 (2023).

13. Togo, A. First-principles Phonon Calculations with Phonopy and Phono3py. *J. Phys. Soc. Jpn.* **92,** 012001 (2023).

14. Alkauskas, A., Buckley, B. B., Awschalom, D. D. & de Walle, C. G. V. First-principles theory of the luminescence lineshape for the triplet transition in diamond NV centres. *New Journal of Physics* **16,** 073026. https://dx.doi.org/10.1088/1367-2630/16/7/073026 (July 2014).

15. Tawfik, S. A. & Russo, S. P. PyPhotonics: A python package for the evaluation of luminescence properties of defects. *Computer Physics Communications* **273,** 108222. ISSN: 0010-4655. https://www.sciencedirect.com/science/article/pii/S0010465521003349 (2022).

16. Lee, J. H. *et al.* Strong Zero-Phonon Transition from Point Defect-Stacking Fault Complexes in Silicon Carbide Nanowires. *Nano Letters* **21,** 9187–9194. ISSN: 1530-6984. https://doi.org/10.1021/acs.nanolett.1c03013 (Nov. 2021).
